# Supplementary material for: Simulating the Photochemical Birth of the Hydrated Electron in Liquid Water
Source: Nat Commun. 2026 Mar 10;17:3764. doi: 10.1038/s41467-026-70045-7 (PMC13106769; doi:10.1038/s41467-026-70045-7)
Supplement: Supplementary file 1 — Supplementary Information [file 41467_2026_70045_MOESM1_ESM.pdf]

Supplementary Information for: Simulating the Photochemical Birth of the Hydrated Electron in Liquid Water

Gonzalo Díaz Mirón[1*], Cesare Malosso[2,3], Solana Di Pino[1], Colin K. Egan[4], Diganta Dasgupta[1,2], Christopher J. Mundy[5,6], Ali Hassanali[1*]

[1]Condensed Matter and Statistical Physics, The Abdus Salam International Center for Theoretical Physics, 34151 Trieste, Italy.
[2]SISSA – Scuola Internazionale Superiore di Studi Avanzati, Trieste 34136, Italy.
[3]Laboratory of Computational Science and Modeling, IMX, École Polytechnique Fédérale de Lausanne, 1015 Lausanne, Switzerland.
[4]Initiative for Computational Catalysis, Flatiron Institute, New York 10010, USA.
[5]Physical and Computational Sciences Directorate, Pacific Northwest National Laboratory, Richland, Washington 99354, USA.
[6]Department of Chemical Engineering, University of Washington, Seattle, Washington 98195, USA.

*Corresponding author(s). E-mail(s): gdiaz_mi@ictp.it; ahassana@ictp.it;

Suppl. Note 1. Comparison between ROKS and TDDFT for the initial Photo-absorption

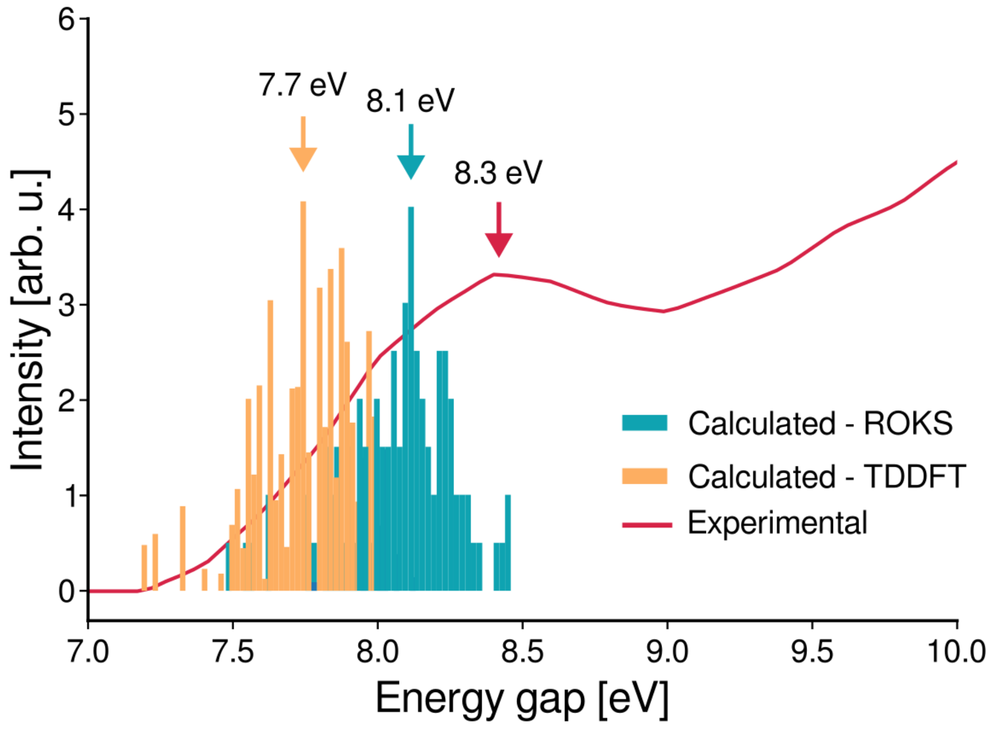

Fig. 1 Initial photo-absorption in liquid water: The histogram of energy gaps calculated with ROKS is shown as light-blue bars, while the histogram of energy gaps weighted by oscillator strength from TDDFT calculations is presented as orange bars. Both theoretical spectra were scaled (while preserving relative intensities) to better visibility and comparison with the experimental absorption spectrum, depicted as a red line. The experimental spectrum was digitalized from Ref **?** . Source data are provided as a Source Data file.

Suppl. Note 2. Partitioning of the electronic spin density

The partitioning of the spin density is a key variable used in our approach to identify the water molecules that are involved in the photoexcitation in an agnostic manner. However, this approach uses an input parameter namely, the cutoff radius. Supplementary Figure 2 shows the IPR histograms (same to the one presented in Figure 1**B** in the main text) using different parameters. We can see that the precise numbers of water molecules involved are different. Nonetheless, they all follow the same physical

trends: most of the excitation are localized on a single water molecules and a small contribution of the delocalized excitation can be observed.

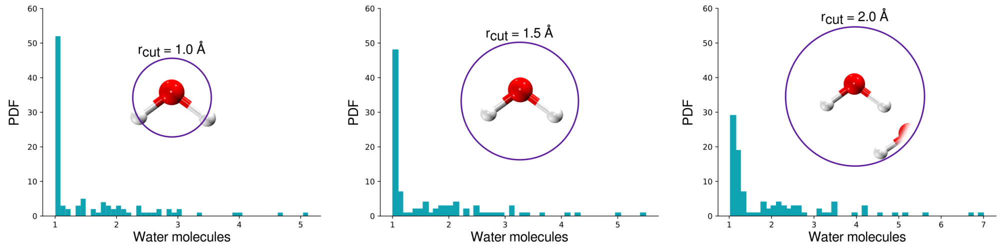

Fig. 2 Validation of the cutoff radius used in the decomposition of the electronic spin densities into the water molecules. Source data are provided as a Source Data file.

When using a larger cutoff value ($r_{cut} = 2.0$ Å), the partitioning begins to include contributions from neighboring water molecules, leading to an overestimation of the degree of delocalization. Cutoff values of 1.0 and 1.5 Å yield slightly different numerical results. However, since both provide consistent physical conclusions, we chose to use a cutoff of 1.0 Å for the analysis presented in the manuscript.

Suppl. Note 3. Hydrogen Bond Network in the Photo-absorption

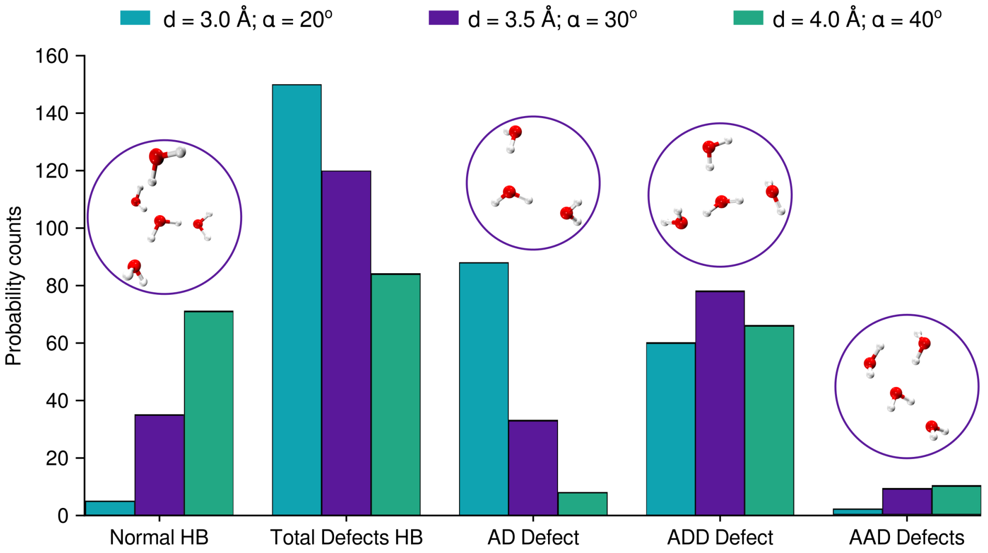

Fig. 3 Validation of the distance and angle parameters employed for the study of the Hydrogen Bond Network of the water molecules involved in the photo-excitation. Parameters presented as a purple bars are the ones used in the main text. Source data are provided as a Source Data file.

Suppl. Note 4. Time decay of the mechanisms

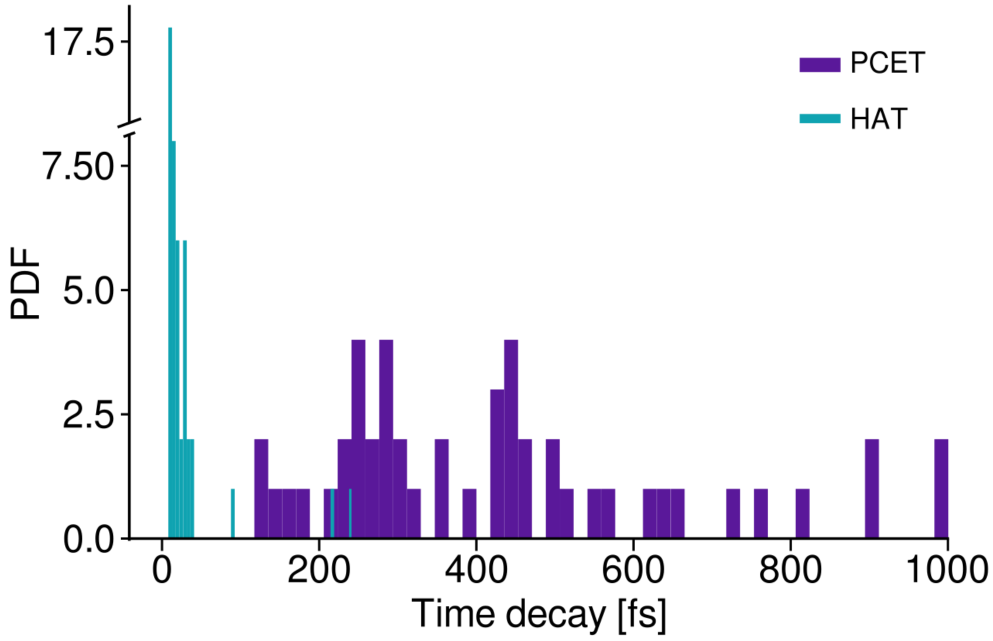

Fig. 4 Probability Distribution Function (PDF) of the time decays for PCET mechanism in purple bars and for the HAT process in light-blue bars. Source data are provided as a Source Data file.

Suppl. Note 5. Overcoming the issue on the electron center calculation

In this section, we address one of the key challenges discussed in the manuscript namely the proper identification of the excess electron. Supplementary Figure 5 presents the spin densities for two representative frames, each corresponding to one of the two mechanisms observed in the photochemistry of water.

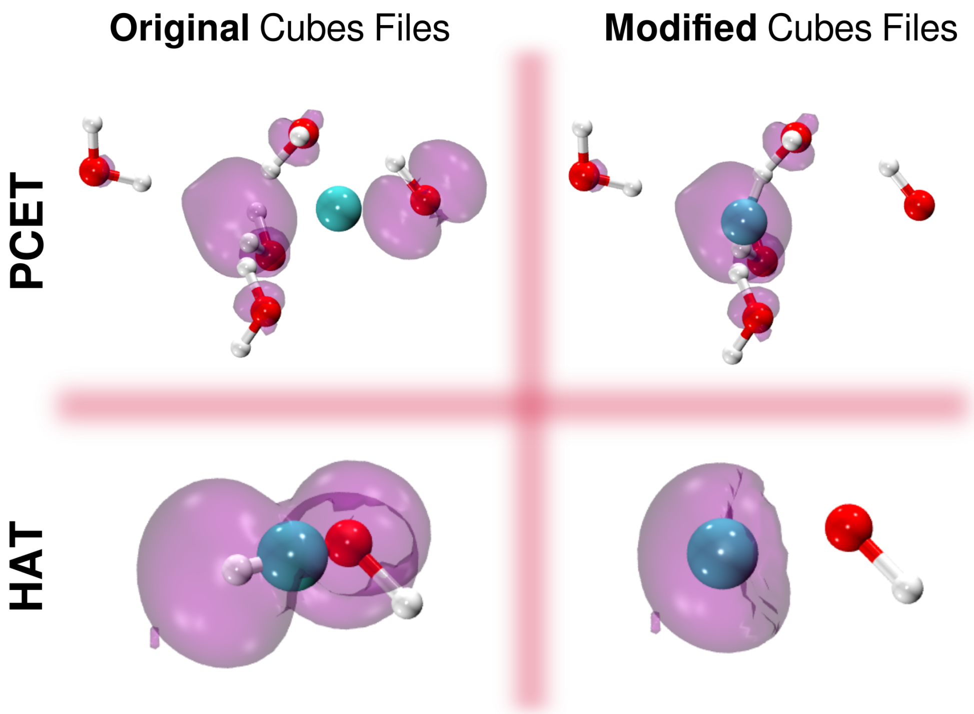

Fig. 5 Identification of the excess electron. Original cubes files are shown in the left panels (as obtained from the CP2K software using ROKS method). The Modified cube files obtained as an output of our procedure are shown in the right panels. The center of the spin density for each kind of cube files are represented as a blue ball. We show the cube files for a selected frame exhibiting both PCET (upper panels) and HAT (lower panels) mechanisms.

As shown in the left panels of Supplementary Figure 5, calculating the electron center using the original cube files results in a position (indicated by a light-blue sphere) located approximately in the middle between the $HO^{\bullet}$ and the hydrated electron (top-left panel) or the $H^{\bullet}$ (bottom-left panel). To address this issue, our protocol, described in the manuscript, generates a modified cube file. This improved representation, shown in the right panels of Supplementary Figure 5, allows for a more physical determination of properties such as the electron center and the radius of gyration.

Suppl. Note 6. Identifying the $HO^{\bullet}$ species

In all trajectories, we observed that the dissociating species originates from the water molecule involved in the initial photo-absorption event. However, in cases where the excitation is delocalized (see Figure 1**B** in the main manuscript), it is not straightforward to determine in advance which specific molecule will dissociate. To automate this process, we first identified the two hydrogen atoms bonded to each oxygen at time $t = 0$. We then calculated the longest OH bond distances for each water molecule.

Supplementary Figure 6**A** shows the longest bond distance for each water molecule for a representative trajectory. It is evident that only one water molecule undergoes dissociation. This behavior is consistently observed across the full ensemble of trajectories, as shown in Supplementary Figure 6**B**, where we plot the average maximum OH bond length for each water molecule across all excited state trajectories. This approach provides a simple and reliable method for identifying the water molecule that forms the HO$^{\bullet}$. Additional confirmation of the HO$^{\bullet}$ is presented in the next section, where we quantify the amount of electron density carried by this species.

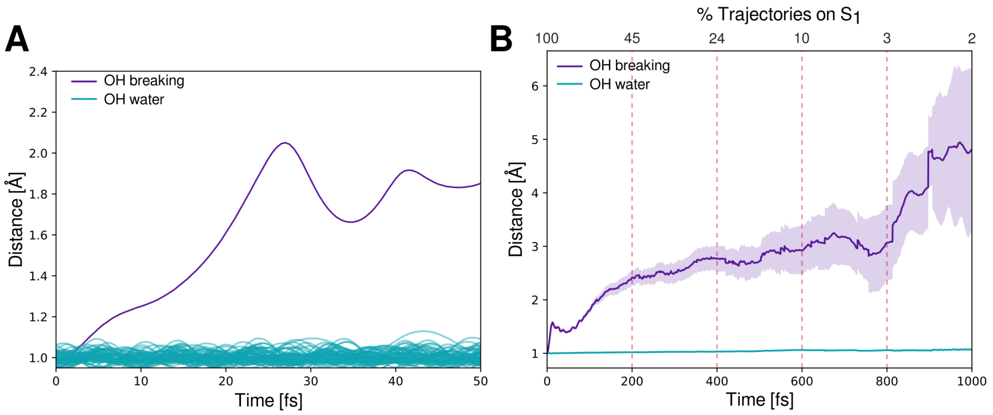

Fig. 6 Identification of the hydroxyl radical. **Panel A**: shows the longest OH bond distances for all the water molecules in a representative trajectory on the excited state. Purple line represent the longest OH bond distance that is breaking, which can be identified within the 10 fs of the simulation. Green lines are the longest OH bonds of the remaining water. **Panel B**: shows the average longest OH bond distances for the molecule whose water molecule dissociates (purple line) as well as the other waters (green line). The transparent shadow represent the standard error (SE). The upper axis shows the percentage of the entire ensemble of trajectories that remain on the S_1 potential at each time during the simulation. The SE was calculated using $SE = \frac{\sigma}{\sqrt{N}}$ with the data coming from an analysis of 100 trajectories. The error bars reported represent those coming from trajectories that remain in the S_1 state. Source data are provided as a Source Data file.

Suppl. Note 7. Removing spin density contributions from the HO$^{\bullet}$

Supplementary Figure 7 shows the probability density distribution function of the amount of the electron that is essentially removed from the candidate HO$^{\bullet}$ species across all the ensemble of trajectories. The distribution exhibits a peak very close to 1, confirming that 1 electron is removed from a species that is the HO$^{\bullet}$.This result also confirms the presence of a radical rather that the ion (OH^-) in the simulation. After the removal of the spin density around the radical we generated the modified cube file presented in the right panels in Supplementary Figure 5. Using this modified cube files, we determined the electron center and radius of gyration.

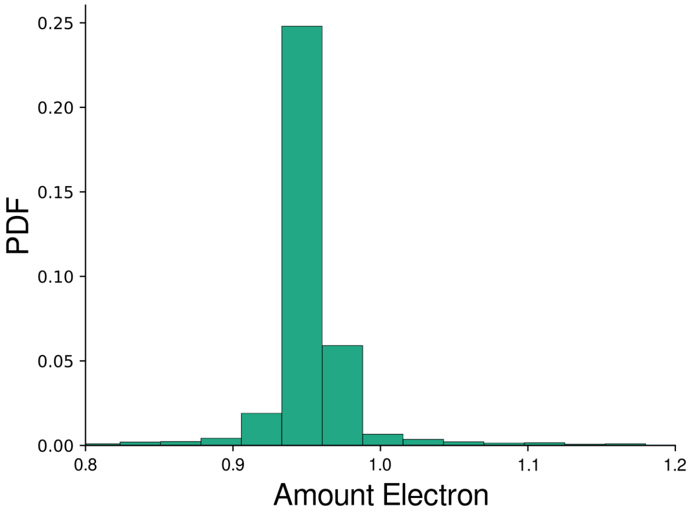

Fig. 7 Probability Distribution Function of the amount of the electron that is removed from the $HO^{\bullet}$ using all the trajectories. Source data are provided as a Source Data file.

Suppl. Note 8. Electron Center and Gyration radius calculation

Using the modified cube files for the spin density (right panels in Supplementary Figure 5) we can now determine the electron center and radius of gyration using the following expressions[1]:

$$r_c = \int \rho^s(\mathbf{r})\mathbf{r}d\mathbf{r} \tag{1}$$

$$\mathbf{S} = \int (\mathbf{r} - r_c)(\mathbf{r} - r_c)\rho^s(\mathbf{r})d\mathbf{r} \tag{2}$$

$$r_g = \sqrt{\lambda_1^2 + \lambda_2^2 + \lambda_3^2} \tag{3}$$

where ρ^s, r_c, λ_i and r_g represent the spin density, electron center, the eigenvalues of the second moment tensor $\mathbf{S}$ and the gyration radius, respectively. It is important to note that the previous definitions are not strictly valid under periodic boundary conditions[1]. To overcome this, we follow the approach proposed in previous studies[1], in which the cube files are re-centered on the maximum of the spin density. This procedure ensures that the spin density decays to zero (or to very small values) at the edges of the simulation box, leading to more numerically stable results.

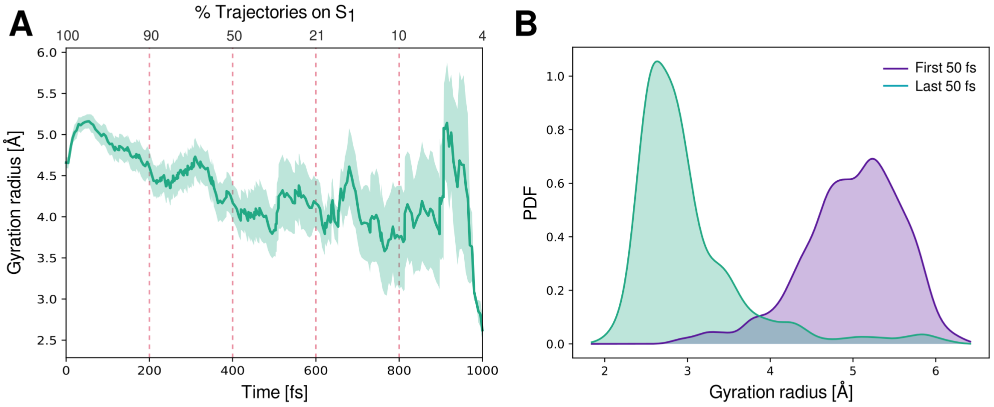

Fig. 8 Gyration radius of the hydrated electron. **Panel A:** Time evolution of the average gyration radius (solid line) using all the trajectories that undergo the PCET mechanism, standard error (SE) is represented as transparent shadow. Upper axes represents the percentage of trajectories that remain in the S_1 potential energy surface. **Panel B:** Probability Density Function (PDF) of the gyration radius using the first 50 fs after the photo-absorption (purple) and the last 50 fs, before the non-radiative decay (green), using all the trajectories that exhibit the PCET mechanism. The SE was calculated using $SE = \frac{\sigma}{\sqrt{N}}$ with the data coming from an analysis of 100 trajectories. The error bars reported represent those coming from trajectories that remain in the S_1 state. Source data are provided as a Source Data file.

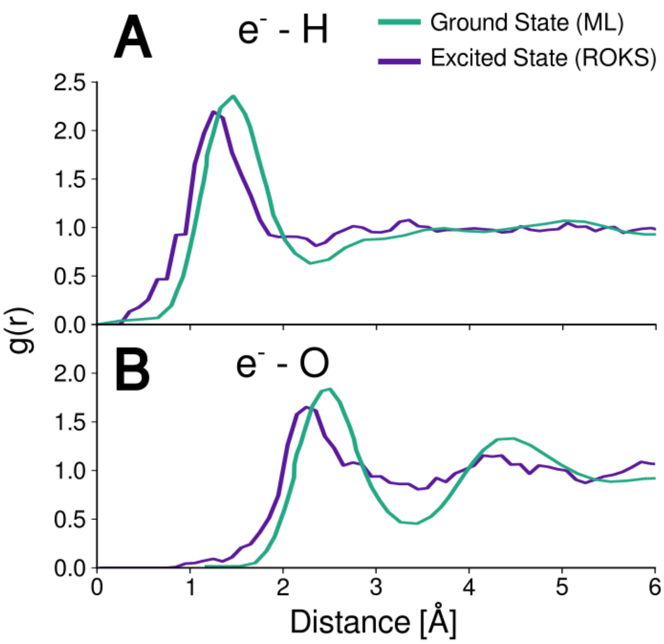

Fig. 9 Radial Distribution Function of the hydrated electron. **Panel A:** shows the the radial distribution function $g(\mathbf{r})$ between the electron and the hydrogen atoms of the waters. **Panel B:** shows the $g(\mathbf{r})$ for the electron and the oxygen atoms of the waters. The results obtained on the excited state with the ROKS method are shown in purple. The results using a recently reported machine-learning (ML) potential from Ref1 are shown in green. All the results for the excited state are for the case of low gyration radius (lower than 4 Å, see main text). Source data are provided as a Source Data file.

Suppl. Note 9. Validation of functional and basis set in ROKS approach

The main objective of this section is to assess the sensitivity of the ROKS approach to variations in the basis set and exchange–correlation functional. To this end, we selected two representative trajectories: one corresponding to the HAT mechanism and one to the PCET mechanism, originally computed using the double-zeta basis set (DZVP)[2] and the PBEh(40)-rVV10 functional[3] here referred to as PBEh (additional details of the calculation are provided in Methods section). These trajectories were then recalculated using a triple-zeta basis set (TZV2P)[2] and the CAM-B3LYP functional[4]. To reduce computational cost, the PCET trajectory was recomputed at 5 fs intervals, while the HAT trajectory was recalculated at every time step. The results are summarized in Supplementary Figure 10.

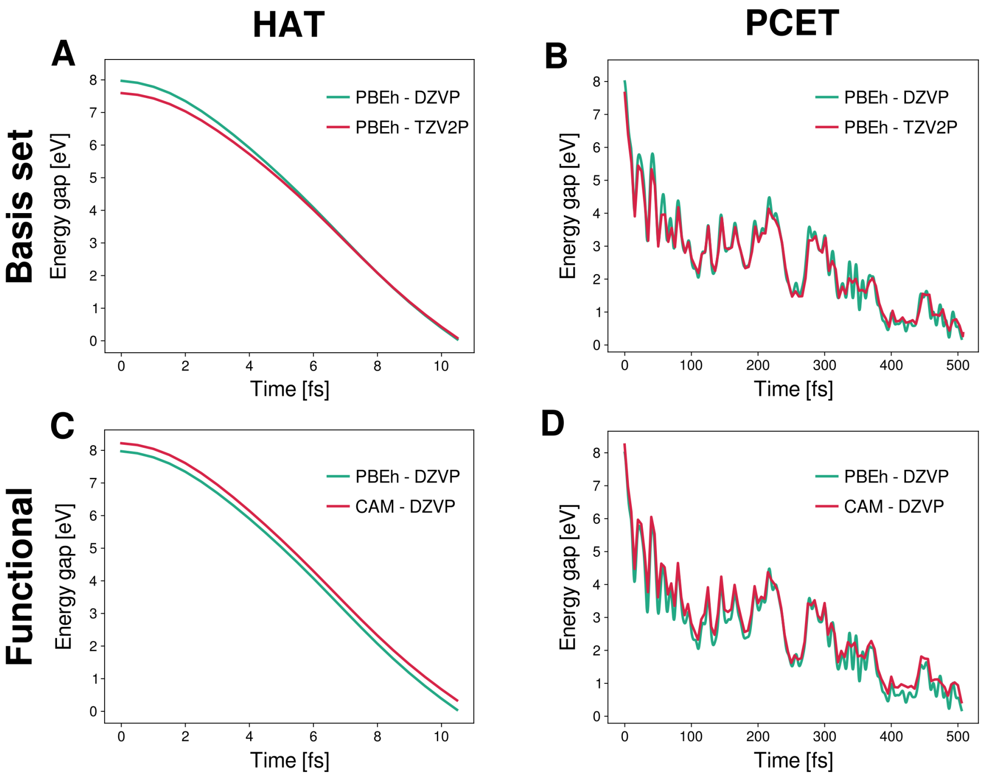

Fig. 10 Basis set and exchange-correlation functional validation within ROKS framework. Comparison between the DZVP (green line) and TZV2P (red line) basis sets using the PBEh(40)-rVV10 functional (PBEh) is shown in **Panel A** and **Panel B** for the HAT and PCET mechanisms, respectively. Similarly, comparison between the PBEh (green line) and CAM-B3LYP (CAM, red line) functionals using the DZVP basis set is shown in **Panel C** and **Panel D** for the HAT and PCET mechanisms, respectively. For the PCET trajectory, the PBEh-TZV2P (red line in **Panel B**) and CAM-DZVP (red line, **Panel D**) calculations were permorfed at configurations sampled every 5 fs to reduce the computational cost. Source data are provided as a Source Data file.

Suppl. Note 10. Incorporating Non-Adiabatic Effects into the Photochemistry of Liquid Water

In this work, we employed an energy gap threshold to determine whether the system remains in the excited state or undergoes a non-radiative transition to the electronic ground state. This methodology is widely used in non-adiabatic dynamics when non-adiabatic coupling vectors (NACVs) are not available[5, 6], as is the case in the present ROKS framework. To further assess the robustness of our approach, we additionally computed the Landau–Zener (LZ) probabilities associated with the non-radiative $S_1 \rightarrow S_0$ transition. Unlike the simple energy gap criterion, the LZ approach incorporates both the energy gap and the curvature of the potential energy surfaces (see equation below):

$$P_{ij}^{LZ} = exp\left(-\frac{\pi}{2\hbar}\sqrt{\frac{\Delta E_{ij}^3}{d^2\Delta E_{ij}/dt^2}}\right) \quad (4)$$

where ΔE_{ij} is the energy gap between the states i and j.

This method has been shown to closely reproduce results obtained from simulations that explicitly include NACVs across a wide range of systems and electronic structure levels[7–11].

Supplementary Figure 11 presents the evolution of the LZ probabilities and the energy gap for representative trajectories of the HAT (**Panel A**) and PCET (**Panel B**) mechanisms. In addition, **Panel C** shows a 2D density plot of the LZ probabilities versus the energy gap for all trajectories analyzed in this work.

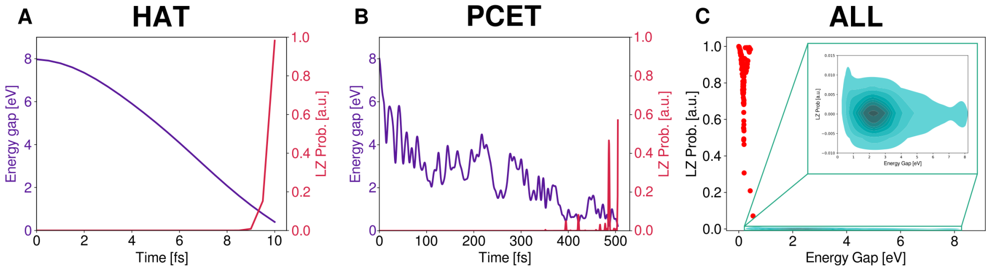

Fig. 11 Landau-Zener (LZ) Probabilities. Energy gap (purple line) and LZ probabilities (red line) as a function of time for representative HAT (**Panel A**) and PCET (**Panel B**) trajectories. **Panel C:** shows a 2D density plot of the LZ probabilities versus energy gap for all trajectories, with the inset providing a zoomed-in view at small LZ values. Red points indicate the values at the $S_1 \to S_0$ crossing events. Source data are provided as a Source Data file.

As shown in Supplementary Figure 11, the LZ probabilities increase sharply when the energy gap becomes small, confirming the consistency and validity of our energy gap based criterion for both mechanisms. For the ensemble of trajectories considered in this work, the majority of non-radiative transitions occur at high LZ probability values, as highlighted by the red points in Supplementary Figure 11**C**.

References

[1] Lan, J., Kapil, V., Gasparotto, P., Ceriotti, M., Iannuzzi, M., Rybkin, V.V.: Simulating the ghost: quantum dynamics of the solvated electron. Nature communications **12**(1), 766 (2021)

[2] VandeVondele, J., Hutter, J.: Gaussian basis sets for accurate calculations on molecular systems in gas and condensed phases. The Journal of chemical physics **127**(11) (2007)

[3] Vydrov, O.A., Van Voorhis, T.: Nonlocal van der waals density functional: The simpler the better. The Journal of chemical physics **133**(24) (2010)

[4] Yanai, T., Tew, D.P., Handy, N.C.: A new hybrid exchange–correlation functional using the coulomb-attenuating method (cam-b3lyp). Chemical physics letters **393**(1-3), 51–57 (2004)

[5] Crespo-Otero, R., Barbatti, M.: Recent advances and perspectives on nonadiabatic mixed quantum–classical dynamics. Chemical reviews **118**(15), 7026–7068 (2018)

[6] Prlj, A., Taylor, J.T., Janoš, J., Slavíček, P., Agostini, F., Curchod, B.F.: Best practices for nonadiabatic molecular dynamics simulations. arXiv preprint arXiv:2508.05263 (2025)

[7] Suchan, J., Janoš, J., Slavicek, P.: Pragmatic approach to photodynamics: Mixed landau–zener surface hopping with intersystem crossing. Journal of Chemical Theory and Computation **16**(9), 5809–5820 (2020)

[8] Tokić, N., Piteša, T., Prlj, A., Sapunar, M., Došlić, N.: Advantages and limitations of landau-zener surface hopping dynamics. Croatica chemica acta **97**(4), 1–11 (2024)

[9] Xie, W., Domcke, W.: Accuracy of trajectory surface-hopping methods: Test for a two-dimensional model of the photodissociation of phenol. The Journal of Chemical Physics **147**(18) (2017)

[10] Díaz Mirón, G., Lien-Medrano, C.R., Banerjee, D., Morzan, U.N., Sentef, M.A., Gebauer, R., Hassanali, A.: Exploring the mechanisms behind non-aromatic fluorescence with the density functional tight binding method. Journal of Chemical Theory and Computation **20**(9), 3864–3878 (2024)

[11] Martinka, J., Zhang, L., Hou, Y.-F., Martyka, M., Pittner, J., Barbatti, M., Dral, P.O.: A descriptor is all you need: Accurate machine learning of nonadiabatic coupling vectors. The Journal of Physical Chemistry Letters **16**, 11732–11744 (2025)
